# Adenosinergic axis and immune checkpoint combination therapy in tumor: A new perspective for immunotherapy strategy

**DOI:** 10.3389/fimmu.2022.978377

**Published:** 2022-09-08

**Authors:** Zhaoyun Liu, Xiaohan Liu, Hongli Shen, Xintong Xu, Xianghong Zhao, Rong Fu

**Affiliations:** Department of Hematology, Tianjin Medical University General Hospital, Tianjin, China

**Keywords:** adenosine, CD39, CD73, adenosine receptor 2A, PD-1, CTLA- 4

## Abstract

There are two figures and one table in this review, the review consists of 5823 words, without the description of figures and table, but including references.

Tumor cells escape anti-tumor immune responses in various ways, including functionally shaping the microenvironment through the secretion of various chemokines and, cytokines. Adenosine is a powerful immunosuppressive metabolite, that is frequently elevated in the extracellular tumor microenvironment (TME). Thus, it has recently been proposed as a novel antitumor immunoassay for targeting adenosine- generating enzymes, such as CD39, CD73, and adenosine receptors. In recent years, the discovery of the immune checkpoints, such as programmed cell death 1(PD-1) and cytotoxic T lymphocyte antigen 4 (CTLA-4), has also greatly changed treatment methods and ideas for malignant tumors. Malignant tumor immunotherapy has been developed from point-to-point therapy targeting immune checkpoints, combining different points of different pathways to create a therapy based on the macroscopic immune regulatory system network. This article reviews the theoretical basis of the adenosine energy axis and immune checkpoint combined therapy for malignant tumors and the latest advances in malignant tumors.

## Adenosinergic axis and tumor immunology

**Figure 1:**
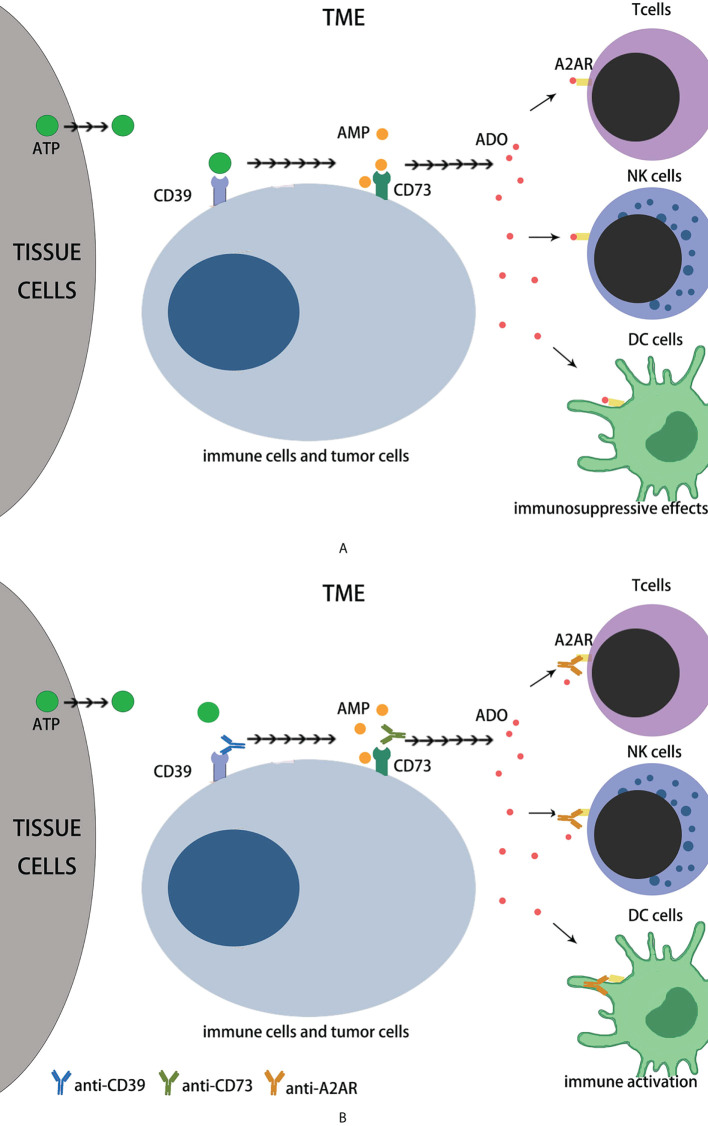
The immune effects of adenosine axis in TME. **(A)** The eATP can be released in large quantities during cell necrosis, apoptosis and mechanical damage, and can be actively secreted by tumor cells and other cells in the TME, which is triggered by hypoxia, chronic inflammation, nutrient deprivation, or cytotoxic drugs. Extracellular ATP is broken down by CD39 to AMP, then CD73 to adenosine. Extracellular adenosine binds to A2AR on immunocytes such as T cells, NK cells and DC cells, inhibiting their immune function. **(B)** Role of monoclonal antibodies in adenosine axis. CD39 mAb prevents eATP from binding to CD39 to reduce AMP production; CD73 mAb prevents AMP from binding to CD73 to reduce ADO production. A2AR mAb prevents ADO from binding to A2AR and inhibits its immunosuppressive effect.

Adenosine is an important regulator of metabolism and a key immune checkpoint regulator associated with tumors evading the host immune system (1–4). Extracellular adenosine (eADO) inhibits immune function. One of the major mechanisms of tumor immune evasion is the production of high eADO levels *via* the overexpression of ectonucleotidases (5–7). An effective immunosuppressive microenvironment is sustained when ADO functions synergistically or in combination with other immunosuppressive mechanisms (8). In 2006, high extracellular adenosine levels in tumors were discovered to play a key role in evading antitumor immune responses (9), and an environment rich in adenosine in the tumor may induce incompetent T cells (1, 10–12). The adenosine pathway is currently considered important for the effectiveness of immunotherapy and has become an important target for cancer therapy (1, 13).Endogenous ATP (eATP) can be released in large quantities through cell necrosis, apoptosis and mechanical damage (14), and can also be actively secreted by tumor cells, immunocytes, and other histocytes in the TME, triggered by various cell damage factors such as hypoxia, chronic inflammation, and cytotoxic drugs (2). The main source of eADO is the continuous degradation of eATP, which involves many different extracellular enzymes, including NTPDase1/CD39 and CD73 (2, 13, 14). CD39 is highly expressed in the tumor endothelium of the TME and on most immunocytes (including macrophages, myeloid cells, and FOXP3+ regulatory T cells (Treg) (3). The CD39 topological domain consists of two transmembrane domains, including short cytoplasmic N-and C-terminal segments and a large extracellular hydrophobic domain containing the active site (15).The extracellular domain contains five conserved propyrylase regions from ACR1 to ACR5, among which the amino acid sequences of ACR1 and ACR5 contain phosphate-binding motifs, which are believed to be critical for stabilizing the interaction between the enzyme and its nucleotide substrate during phosphate cleavage (4). CD39 can stabilize FOXP3+Tregs, contribute to their immunosuppressive function (16), promote type I Treg differentiation, produce IL-10, and restrict the activation of NLRP3 inflammatory bodies in dendritic cells (DCs) (2, 13, 14). CD73 can be found in different kinds of tissues, which includes the colon, liver, kidney, brain, lungs, and heart; leukocytes and endothelial cells of peripheral blood, lymph nodes, spleen and bone marrow (3). CD73 is now known as glycosyl phosphatidyl inositol (GPI)-anchored protein (17), which is a homodimeric disulfide linker protein of 548 amino acids, of which the N-terminus provides a binding site for two catalytic divalent metal ions and the C-terminus is a binding site for AMP (18). The expression and function of CD73 are elevated in the presence of hypoxia and inflammatory mediators (TGF- B, IFNs, TNF-α, IL-1B, PGE2, etc.), and the expression of CD73 is also increased in several tumor tissues, suggesting that CD73 is involved in tumor genesis and development (2, 13, 14, 19), eATP is decomposed into eADO through the sequence of CD39 and CD73, which bind to adenosine receptors on the cell membrane surface (2, 13, 14). Among several known adenosine receptors, adenosine receptor A2a (A2aR) is the predominant subtype and is mainly expressed in immunocytes (20). A2aR stimulation usually provides immunosuppressive signals that inhibit T cell proliferation, cytotoxicity, cytokine production, NK cell cytotoxicity, NKT cell cytokine production, CD40L upregulation, macrophage/DC antigen presentation, etc. (1, 19) (**Figure 1**). Based on the above background analysis, when we want to antagonize the immunosuppressive effect of eADO in the TME, we can start from the following three aspects: 1. It reduces the expression of CD39 in tumor cells and inhibits the conversion of eATP to AMP; 2. It reduces the expression of CD73 molecules on tumor cells and blocks the conversion of AMP into eADO, thereby reducing the binding of eADO to A2aR receptors on immune cells; 3. It reduces the expression of A2aR in immune cells, making them unable to combine with eADO to maintain their normal immune function.

## Immune checkpoint

**Figure 2:**
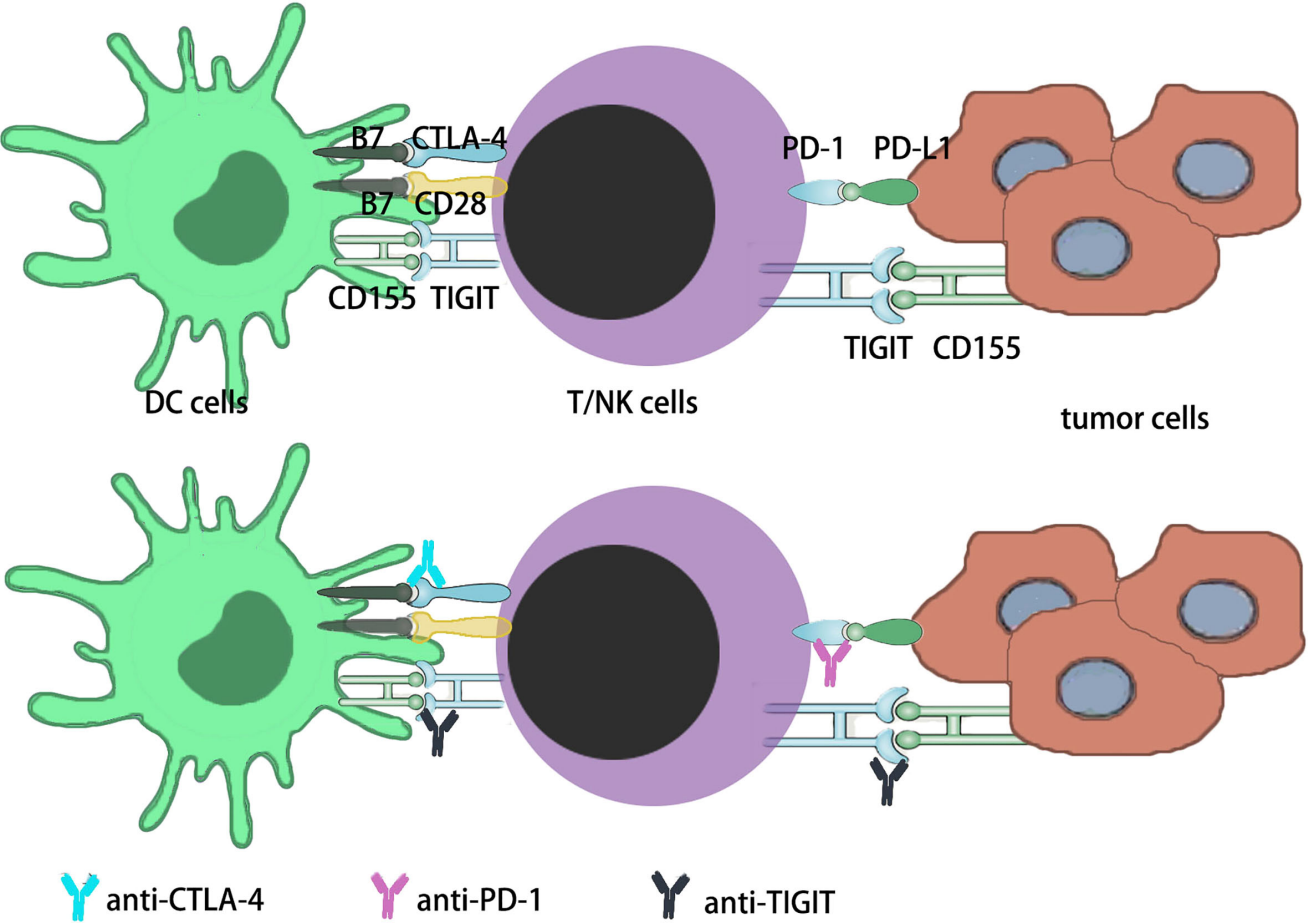
Immune checkpoints in current studies. i) The PD-1 is expressed on activated T cells in the early-stage lymph node as well as late-stage tumor tissues in the TME. In the early stage and late stage, PD-1 sustains immune homeostasis by decreasing activated T cells function. Tumors might become resistant to this suppression signaling, increasing the survival potential of the tumor cells. ii) The CTLA-4 is expressed on T cells that are activated by DCs in the lymph node. By MHC interaction with T cell receptor and B7 signal interaction with CD28 on T cells. In order to sustain immune homeostasis, CTLA-4 downregulates the function of activated T cells through the interaction of B7 signaling with CTLA-4 on T cells. Tumors may develop toleration to this inhibitory signal, thus improving the survival potential of tumor cells. iii) TIGIT is expressed both on NK cells and T cells, which includes CD4+ T cells, CD8+ T cells and Tregs. TIGIT has three ligands, CD155, CD112 and CD113, and the main ligand for TIGIT is CD155. The main effect of TIGIT is downregulating the function of NK cells and T cells.

The immune system consists of innate and acquired immunity, which, once activated, clears infectious pathogens and tumor cells. Inhibitory pathways in antimicrobial or antitumor immune responses normally maintain auto-tolerance to avoid excessive damage and limit associated tissue damage (21, 22). This receptor and ligand inhibitory pathways are known as “Immune Checkpoint” and are used by tumor cells to avoid Immune attack. The development of monoclonal antibodies to inhibit these checkpoints, thereby removing the Inhibition of immunocytes and enabling them to recognize and kill tumor cells, is called “Immune Checkpoint Inhibition”. These drugs are called “ immune checkpoint inhibitors” (ICI) (23, 24). FDA-approved anti-CTLA-4 and anti-PD-1 antibodies for cancer treatment, which led to the belief that immunotherapy for cancer was realistic and further encouraged the development of other new ICIs (25–27). Immunotherapy is becoming an important treatment for cancer patients (1, 13). Immune checkpoint blocking (ICB) based on monoclonal antibody (mAb) has also proven to be a safe and effective treatment for hematologic malignancies in the past decade (22, 27). In oncology, checkpoints currently targeted by inhibitors to amplify the reactivity of T cells, NK cells and bone marrow cells include CTLA-4 (28), PD-1, PD-L1 (PD1 ligand 1/CD274),LAG-3 (CD223), TIM3 (T cell immunoglobulin-3), TIGIT(T cell immunoglobulin and ITIM domain) (29), VISTA (V-domain immunoglobulin suppressor of T cell activation) (30), B7/H3(CD276), KIR (killer cell immunoglobulin-like receptors),NKG2A, A2AR, CD39, CD73, CSF1R, and CD47 (22, 31, 32) (**Figure 2**).

Each member of the adenosine signaling pathway constitutes a different drug target, meaning that it is possible for combined therapy with more than one drug to target this or complementary signaling pathway (33). Many of these combinations are currently in preclinical and clinical trials, such as anti-CD73 and anti-A2aR combinations, anti-CD73 and anti-PD-1 combinations, and anti-A2aR and anti-TIGIT antibody combinations (21, 31, 34–36) (**Table 1**).

**Table 1:** Application of different targets on adenosinergic axis combined with various immune checkpoints in tumor therapy.

*Adenosine axis*	*ICIs*	*Disease*	*Experimental subjects*	*Mechanism*	*References*
*CD39*	PD-1/PD-L1	Melanoma Fibrosarcoma HCC NSCLC	Mouse model Human tissue, Mouse model Human tissue	Restored T cell proliferation Enhance CD8+T cells’ activation Co-expression was detected	(11) (37) (38)
	CTLA-4	Melanoma HNSCC	Mouse model Human tissue	Enhance NK cells’ activation Co-expression was detected	(39) (40)
	TIGIT	AML	Human tissue	Enhance T/NK cells’ activation	(41, 42)
*CD73*	PD-1/PD-L1	Breast cancer Colon carcinoma Prostate carcinoma Fibrosarcoma Rectal cancer	Mouse model Human tissue Mouse model. Cell line Cell line Cell line Cell line Mouse model	Enhance T/NK cells’ activation Enhance levels of secreted IFNγ and TNFα Enhance CD8+T cells’ activation Enhance CD8+T cells’ activation Enhance CD8+T cells’ activation	(43, 44) (24, 45) (24) (24) (46)
	CTLA-4	Colon carcinoma Prostate carcinoma Melanoma	Cell line Cell line Mouse model	Enhance CD8+T cells’ activation Enhance CD8+T cells’ activation Enhance T cells’ activation and levels of secreted IFNγ	(24) (24) (47)
*A2aR*	PD-1/PD-L1	Melanoma RCC Breast cancer	Human T cell Mouse model Human tissue Clinical trial Cell line and mouse model	Restore T cell activation; enhance T cell activation Associated with tumor prognosis Enhance CAR T cell efficacy and enhance the IFNγ production	(10, 48, 49) (50, 51) (49, 52, 53)
	CTLA-4	Colon carcinoma	Cell line and mouse model	Enhance survival, proliferation of T cells	(54)
	TIGIT	AML OAC	Human tissue Human tissue	Enhance T/NK cells’ activation As evaluation standard of chemotherapy regimen	(42) (55)

## Combination of CD39 with immune checkpoints

The rapid development of flow cytometry in recent years has further confirmed the expression of CD39 in tumor cells, particularly in melanoma, lymphoma, and chronic lymphocytic leukemia (CLL) cell lines (13, 14). In melanoma B16F10 mouse model and colorectal cancer Mc-38 mouse model, CD39-defective mice were resistant to tumor metastasis (31, 56). It has been documented that all cells expressing CD39 exhibit strong ATPase activity, which can be counterbalanced by CD39 inhibitors, such as ARL-67156 and POM-1, by measuring the degradation of eATP or releasing free phosphate from the cell culture supernatant. Treatment with BY40, a CD39 blocking antibody currently under preclinical development, reduces the inhibition of CD4+ and CD8+T cell proliferation, which is induced by tumor tissue and increases cytotoxicity mediated by cytotoxic T lymphocytes (CTL) and NK cells (16). At present, many studies have shown that human CD39+CD8+ T cells exhibited draining dysfunction or phenotype gene signature of T cells, including highly expressed inhibitory receptors, PD-1 and CTLA-4 (57). Thus targeted therapy of CD39 combined with other immune binding sites has great significance in the therapy of tumors. Currently, the main CD39 mAb used in clinical research is IPH5201, which blocks the hydrolysis of ATP by a membrane and soluble CD39, thus promoting DC maturation and macrophage activation (11); BY40 has been reported to block membrane-associated, but insoluble, human CD39 enzyme activity, but its clinical efficacy has not been evaluated (11, 58); POM1 is mainly used for experimental studies on mice and cell lines (11).

### CD39 mAb combined with PD-1 mAb

PD-1 encodes immunoglobulin superfamily proteins and is focused on sustaining immune tolerance to autoantigens and preventing autoimmune diseases. PD-L1 is a PD-1 ligand blocking the interaction between the tumor cells expressing PD-L1 and tumor-specific T cells expressing PD-1 using PD-1 or PD-L1 antibodies enhance the cytolytic activity of T cells (25). It has strong therapeutic value and significance in solid tumors and hematologic malignancies (33, 59). However, during immunotherapy, many tumors show resistance to PD-1/PD-L1. The exhibition of resistance by patients might be due to the immunosuppressive TME, where ROS or nitrogen oxides (NO) released by bone marrow-derived suppressor cells (MDSC) tire T cells and no longer recognize tumor cells. PD1 resistance and poor prognosis in hepatocellular carcinoma (HCC) patients are associated with the upregulation of CD39 expression in macrophages, and CD39 can be used as a marker of unfavorable prognosis in HCC patients (37). This significantly improved through combination therapy with CD39 mAb. It has been reported that the therapy combining anti-CD39 and anti-PD1 mAbs can further slow tumor growth and that the inhibition of CD39 enzyme function can make the tumor model with inherent drug resistance sensitive to PD1 antibody (11, 38).This may be because CD39 mAb and PD-1 mAb can recover the ability of CD8+T cells to produce cytokines. CD39 mAb combined with PD-1 mAb has become one of the targets of many tumor therapies (37).

### CD39 mAb combined with CTLA-4 mAb

CTLA-4 is a molecule belonging to the immunoglobulin superfamily. It was first discovered in the cDNA libraries of CTLs and expressed in activated T cells, Tregs, and acute myeloid leukemia cells (28, 33). Although CTLA-4 and its homologue CD28 bind to ligand B7 on B cells and APCs, stimulation of CTLA-4 does not result in T cell activation, but rather in T-cell-mediated antibodies that inhibit and prevent allograft rejection (23, 25). Blocking the CTLA-4-B7 interaction with anti-CTLA-4 mAb results in an enhanced alloantigen response that inhibits negative signaling to T cells (32). However, anti-CTLA-4 is rarely effective as a single drug for highly oncogenic and immunogenic tumors. Targeting CD39 with POM-1 has a synergistic effect on anti-CTLA-4 checkpoint blockade. Specifically, blocking CD39 with POM-1 significantly increased the antitumor activation of CTLA-4 mAb in a mouse model of lung metastasis, and showed better efficacy in a CD39-deficient mouse model of tumor transplanted with B16F10 (39). Recent research also showed that the expression of CTLA-4 and CD39 may be potential target molecules that inhibit Treg activity *in situ* (40). Although there is limited literature on the combination of CD39 mAb with CTLA-4 mAb, according to the current study, the combination of the two mAbs has great potential in tumor therapy, especially in the treatment of tumor metastasis.

### CD39 mAb combined with TIGIT mAb

TIGIT is an inhibitory receptor expressed on lymphocytes that has recently attracted attention as the latest target for tumor immunotherapy. This shows the interplay between TIGHT and CD155, which is expressed on APCs or tumor cells, reducing T and NK cell function. TIGIT, a significant inhibitor of antitumor responses, blocks the tumor immune cycle in multiple steps (60–62). Several studies have shown that blocking TIGIT can prevent various solid and hematologic malignancies. In AML, inhibition of CD39 combined with TIGIT can increase AML cell lysis in 2/3 cell lines, and the combined inhibition of TIGIT and CD39 significantly improved NK cell killing activity *in vitro*, thus further enhancing the NK cell killing effect on AML cells (41, 42, 63). Owing to the difference in the expression of the TIGIT/PVRIG axis and CD39 in different NK cell subsets, joint blocking of these pathways may enhance the cytotoxic function of different NK cell subsets *in vivo*. In addition, it has been preliminarily reported that RORγ agonists can simultaneously reduce the expression of CD39, TIGIT, and other immune checkpoints on lymphocytes, and integrate multiple antitumor mechanisms into one therapy. This enhances immune activity and reduces immunosuppression, thus effectively inhibiting tumor growth (64).

## Association of CD73 with immune checkpoints

CD73 is expressed in various types of cancer and is known to promote tumor growth, metastasis, and drug tolerance in glioblastoma, melanoma, leukemia, colon, breast, ovarian, and bladder cancers (13, 19, 65). In human breast cancer cells, high expression of CD73 is related to low response and high resistance to anthracyclines (44, 46, 66). High levels of CD73 are associated with immunosuppression and tumor progression. The overexpression of CD73 in tumors not only leads to metastasis of tumor cells and anthracycline resistance but also leads to immune escape because of excess adenosine production (67, 68). Therefore, inhibitors of CD73 are currently used in combination with existing cancer therapies for cancer immunotherapy, including anti-PD-1/PD-L1 and anti-CTLA-4 therapies (44, 46, 67). Although blocking CD73 alone does not result in a cure, the inhibition of CD73 increases the antitumor effect of immune checkpoint therapies, including anti-CTLA-4 and anti-PD-1 (19, 24, 67). The synergistic effects of combined CTLA-4 mAb with CD73 mAb and combined PD-1 mAb with CD73 mAb immunotherapy have been observed in preclinical models of both breast cancer and colon cancer (24, 45, 68). Currently, MEDI9447(AstrazenecaMedimmune), a human IgG1CD73 mAb (46), can selectively inhibit the activity of CD73ECN and cross-react with mouse and human CD73. MEDI9447 internalized the desetting of CD73 from the cell surface, thereby inhibiting the conversion of AMP to adenosine and removing the inhibition of T cell proliferation mediated by AMP. In an immunoactive mouse tumor model, MEDI9447 reduces immunosuppressive effects and promotes antineoplastic function (45); BMS986179, a high affinity antibody, inhibits the activity of CD73 and mediates the internalization of CD73 (19, 69); CPI-006 (also known as CPX006) acts mainly by inhibiting CD73 activity and/or inducing CD73 downregulation; IPH5301, which blocks AMP from degrading to the immunosuppressant adenosine. At present, these antibodies are undergoing early-stage clinical trials (11, 70).

### CD73 mAb combined with PD-1/PD-L1 mAb

As mentioned above, CD73-derived adenosine strongly mediates tumor immune status and metastasis, and weak patient response to PD-1 antibodies may also be associated with elevated intratomatous adenosine levels. In this context, the combination of CD73, mAb, and PD-1 mAb may be particularly effective in tumor immunotherapy. Currently, various studies have focused on the clinical effects of combining CD73 inhibition with PD-1 blockade. In melanoma, breast cancer, colon cancer, non-small cell lung cancer (NSCLC), prostate cancer, and other malignant tumors (24, 43, 45), combining the CD73 mAb with the PD-1 mAb has shown a more significant effect than these drugs alone. The high expression of CD73 on the surface of tumor cells shows a weaker effect of immunotherapy with a PD-1 antibody, and the combined use of PD-1 mAb and CD73 mAb prominently inhibited tumor growth (46), and increased gene expression related to inflammation and T cell function, causing an increase in the number and activity of tumor-infiltrating CD8+T cells and the production of IFN-γ and TNF-α (20, 46). It has also been reported that MEDI9447 when combined with anti-PD-1 antibodies, can produce a better antitumor effect, which is supported by multiple phases I/II trials based on MEDI9447. Preliminary phase I data for MEDI9447(NCT02503774) have recently been reported (24).The safety of MEDI9447 and duvacizumAb (anti-PD-L1) treatment is controllable, and PD-1 is consistent with its mechanism of function. BMS-986179 was also found to enhance the antineoplastic activity of anti-PD-1mAb in preclinical animal models (19, 71). Notably, the combination of A2aR antagonist and PD-1 antibody also showed an antimetastatic effect. However, the combination therapy with A2aR antagonists was effective only when tumors expressed high CD73 levels, suggesting that CD73 can also be used as a potential tumor indicator to assess the benefit of combination therapy (13, 19, 44).

### CD73 mAb combined with CTLA-4 mAb

The combination of CD73 mAb and PD-1 mAb was more effective against both subcutaneous and metastatic tumors than that of CD73 mAb and CTLA-4 mAb (24). This may be due to the stronger antineoplastic activity of PD-1 mAb itself than that of CTLA-4 mAb or the synergistic effect of CD73 mAb and PD-1 mAb on Tregs. However, CD73 mAb combined with CTLA-4 mAb still has clinical significance and cannot be ignored. CD73 mAb combined with CTLA-4 mAb significantly improved the median survival in a tumor metastasis mouse model (71). In melanoma, the efficacy of anti-CTLA-4 therapy can be enhanced by targeting various immunosuppressive mechanisms in tumor tissues, including CD73. CD73 antibody combined with CTLA-4 mAb significantly inhibited melanoma growth. In a mouse melanoma model, the percentage of infiltrated CD8+T and CD4+T cells significantly increased after the combination of the two antibodies, and the proportion of Tregs was also increased compared with that of the two antibodies alone, which may be due to the increase in CD4+T cells after the combination of the two monoclonal antibodies. At the same time, IFN-γ levels increased in melanoma tissues of mice treated with CD73 antibody in combination with CTLA-4 antibody (47). CTLA-4 mAb is also of great clinical significance in hematologic malignancies such as AML and MDS (22) and has great potential in combination with CD73 antibody in the treatment of malignant diseases of the blood.

## Association of A2AR with immune checkpoints

The A2a receptor (A2aR) in the adenosinergic pathway is an important immune checkpoint. Adenosine levels in the extracellular fluid are increased in the TME because of the special metabolism of tumor cells, which contributes to tumor immune escape. Therefore, A2aR inhibitors are being investigated to enhance the effect of immunotherapy (2, 13). Several A2aR antagonists have been developed and tested in multiple preclinical studies. At least four drugs, CPI-444 (10) PBF-509 (Novartis/Pablobiofarma), MK-3814 (Merck), and AZD4635 (AstraZeneca/Heptares), are currently in phase I clinical trials (13). CPI-444 intensifies antineoplastic immunity and enhances anti-PD-L1 mAb activity in mice. CPI-444 has also been shown to intensify the antitumor effect of adoptive metastases of HER2-specific CD8+T cells in tumor-bearing mice treated with cyclophosphamide and a novel gene-expressed whole-cell vaccine (GVAX) (10). Vipatant (REDOX/Juno therapy) and Etradine (Kyowa Hakko Kirin) are other promising oral A2a antagonists that have previously gone through clinical trials for Parkinson’s disease and may be effective in cancer patients. Recent research has shown that A2aR inhibits T cell proliferation and cytokine secretion and increases the expression of PD-1 and CTLA-4 on the surface (72, 73). Currently, combination therapy with A2aR and other immune checkpoints is also attracting attention (10, 13).

### A2aR mAb combined with PD-1/PD-L1 mAb

As the expression of A2a is increased in antigen-activated T cells and PD-1 is involved in inhibiting T cell function, the combination of targeted blocking of these two molecules is considered a new direction in tumor therapy. Recent clinical studies have shown that in renal cell cancer (RCC) patients, A2aR and PD-L1 expression in the primary tumors may foresee the consequences of therapy with anti-VEGF agents and ICIs (51), and the A2aR antagonist ciforadenant showed monotherapy activity in patients who were resistant to or intractable to previous anti-PD-L1 therapy. Although this trial did not deliberately compare the effects of monotherapy with those of combination therapy, treatment with an A2aR antagonist plus anti-PD-L1 appeared to improve efficacy (50). Studies have shown that blocking A2aR with CPI-444 reduces the expression of checkpoints of various pathways in T-effs and Tregs, including PD-1 and LAG-3. By reducing the expression of immune checkpoints on these T cells, the threshold for anti-PD-1 treatment is lowered. In other words, there is a synergistic reaction with the combination of CPI-444 and PD-1 mAb (10, 48). Moreover, A2aR blockers significantly reduced the expression of PD-1 and LAG-3 in the draining lymph nodes of tumor-bearing mice (53). Another group successfully combined A2aR blockers with anti-PD-1 inhibitors in an anti-tumor regimen in a mouse model (35). Mittal et al. (49) also reported that combining SCH58261, the A2aR inhibitor, with anti-PD-1 therapy significantly reduced the burden of metastasis compared with either monotherapy alone. Uniting therapy with PD-1 mAbs and CPI-444 showed significant improvement in tumor regression and survival in CT26 and MC38 tumor models (more significant in CT26 tumor models) (48). In NSCLC mouse models, A2a receptor inhibition overcomes the resistance of tumor cells to PD-1/PD-L1 blocking treatment. Meanwhile, A2aR and CD73 were upregulated in mice treated with PD-1 or PD-L1 mAbs (52, 53). In mouse models of breast, colon, and hepatocellular carcinomas, drug resistance of tumor cells to PD-1/PD-L1 can be prevented through dual blocking of PD-1 and A2aR. Blocking A2aR after a viral attack also reduced the expression of PD-1, LAG-3, and TIM-3 on CD8+T cells and Tregs. These abundant *in vivo* and *in vitro* experiments suggest that the combination of A2aR blockers and PD-1/PD-L1 antibodies is of great significance in the clinical treatment of tumors (34, 71).

### A2aR mAb combined with CTLA-4 mAb

Combining A2aR mAb CPI-444 with anti-CTLA-4 therapy eliminated tumors in up to 90% of the treated mice, including restoring an immune response in a model against an incomplete response to CTLA-4 monotherapy. Moreover, tumor cells remained suppressed after re-inoculation of mice with tumor cells, suggesting that CPI-444 induces systemic antineoplastic immune memory and that the combination of CPI-444 with CTLA-4 mAb increases the presence of CD8+T cells and IFNγ and Gzm B levels in tumors (71). In a mouse melanoma model, inhibition of both CD73 and A2aR increased CTLA-4 the therapeutic effect. Blocking A2aR plays an important role in regulating T-cell function and significantly reduces melanoma growth (47). Most importantly, the combination of A2aR antagonists and anti-CTLA-4 therapy significantly restricts tumor growth and enhances the antitumor immune response (10, 71). Additionally, other studies have shown that the concomitant blocking of A2aR and CTLA-4 in T cells can synergistically enhance the antitumor response by downregulating PKA, SHP2, and PP2Aα signaling pathways, providing a theoretical basis for A2aR mAb combined with CTLA-4 mAb as a new treatment regimen for tumors (54).

### A2aR mAb combined with TIGIT mAb

The frequency of TIGIT + NK cells in the blood of patients was negatively correlated with AML prognosis. Compared with healthy subjects, AML patients had abnormal NK cell populations in the peripheral blood (PB) and bone marrow (BM), which showed an increased frequency of TIGIT+, PVRIG+, CD39+, and CD69+NK cells. Thus, TIGIT is a potential target for AML treatment (42). The purinergic pathway also regulates NK cell functions. Proliferation and hypoxia of tumor cells increase the utilization of ATP and activate cancer-related CD39 and CD73, which catalyze the continuous dephosphorylation of ATP to AMP and then to eADO. Extracellular adenosine accumulation interacts with adenosine receptors expressed on the surface of NK cells and inhibits signaling through A2aR; therefore, A2aR antibodies are also important targets for tumor therapy (55). It has been demonstrated that the combined blocking of TIGIT and A2aR enhances NK-92 cell-mediated cytotoxicity in AML (42). In other tumors, the combination of TIGIT and A2aR mAbs is still being explored (55).

## Comparison of the efficacy of CD39, CD73, A2AR and immune checkpoint inhibitors in clinical treatment

Studies have shown that A2AR mAb, Ciforadenant (CPI-144) has a good effect on RCC, and its combination with atezolizumab has a better effect than Ciforadenant alone (74). Taminadenant, another A2AR antagonist, has shown promising efficacy in NSCLC, either alone or in combination with the PD-1 mAb, Spartalizumab (75). There does not appear to be a significant difference in the efficacy of A2aR antagonists alone or in combination with immune checkpoint inhibitors for malignancy, but the number of clinical trials in this area is small, and the results of these two trials are not representative. The combination of A2aR antagonists and immune checkpoint inhibitors has been seriously considered in clinical practice and needs to be explored and validated in more clinical trials.

Although there are no cases of CD39 mAb used alone in clinical treatment, clinical studies have shown that the expression of CD39 in chronic lymphocytic leukemia patients is closely related to the stage of disease, the time of first treatment, and the prognosis of patients (76, 77). Similar results have been found in immune-related diseases, such as Crohn’s disease and multiple sclerosis (78, 79). Due to various limitations, treatment with CD39 mAb combined with immune checkpoints such as PD-1, CTLA-4, and TIGIT has been limited to mouse models, cell lines, and human tissues. However, this combination therapy has been proved to have high clinical potential and value by a large number of *in vitro* experiments (11, 37–42).

CD73 is currently mainly used as a prognostic indicator for clinical tumors, including breast and rectal cancers (23, 24, 43–47). Few trials have applied the CD73 mAb in the clinical treatment of tumors. Recent *in vitro* experiments have confirmed that the CD73 mAb can enhance the efficacy of immune checkpoint inhibitors and reduce the formation of resistance. Its combination with immune checkpoint inhibitors is far more effective than either of them alone (23, 24, 43–47).

The efficacy of antagonists of CD39 and CD73, A2aR targets on the adenosinergic axis alone and in combination has been discussed in a study on multiple myeloma (80). *In vitro* experiments confirmed that the inhibitory effect of the three target antagonists alone on myeloma was not as good as that of the two target antagonists combined, while the combined use of three target antagonists, CD39, CD73, and A2aR, had the best inhibitory effect on myeloma.

## Conclusion

In recent years, therapeutic advances in cancer immunotherapy (CIT) have emerged rapidly, reflecting the importance of human immune system interactions with cancer, as well as the complex and highly regulated nature of the immune system (81, 82). In the context of complex immune networks, point-to-point therapy has been unable to achieve satisfactory tumor treatment effects; therefore, combining various targeting axes or immune checkpoints will become a new direction of tumor treatment.

The role of the adenosine axis in the tumor microenvironment is mainly induced by hypoxia; therefore, some studies have also called this the hypoxia–adenosine axis. Extracellular adenosine increases under hypoxic conditions, and simultaneously, the expression of CD39 and CD73 improves (8). Antihypoxia-adenosine therapy is synergistic with other immune checkpoint inhibitors such as CTLA-4 and PD-1 mAbs. The combination of anti-hypoxia-adenosine strategies may enhance the clinical response to other immunotherapies, chemotherapy, and radiotherapy (71). With advances in the treatment of tumors with immune checkpoint blockers such as CTLA-4 and PD-1/PDL1, more therapeutic targets have been sought, including but not limited to the immune targets in the adenosine energy axis mentioned above, to overcome the problems of incomplete tumor regression or recurrence after treatment (3). Immune checkpoint suppressor molecules are emerging as new potential targets for tumor therapy. In the TME, these molecular mechanisms may operate and may be supplementary to approved immunotherapies (26, 83, 84). When the immune escape of tumor cells makes the control of tumors difficult (85), the use of immune checkpoint inhibitors can offset the immune escape of tumor cells to a certain extent and further improve the response rate. Without increasing or even decreasing the adverse events related to excessive tissue damage, autoimmunity, and other immune-associated side reactions associated with the use of immune checkpoint inhibitors alone (22, 86–88). In the future, the treatment trend of malignant tumors will be developed from point-to-point therapy targeting individual immune checkpoints to a combination of immune networks composed of various signaling pathways, such as the adenosine axis. Non-traditional immunotherapies can induce or enhance antitumor immunity. Consequently, they may force tumors to upregulate immune checkpoints, which can be blocked as part of a combined strategy (87, 89–92).

## Author contributions

ZL: Conceptualization, Writing - Review and Editing, Project administration. XL: Writing - Original Draft, Visualization, Resources. XX: Resources. HS: Resources. XZ: Resources. RF: Supervision, Funding acquisition, Project administration. All authors contributed to the article and approved the submitted version.

## Funding

This work was supported by the National Natural Science Foundation of China Youth Project (grant no. 81900131,82000219), the National Natural Science Foundation of China Youth Project (grant no. 8210010129), the Tianjin Municipal Natural Science Foundation (grant no. 18JCQNJC80400, 19JCZDJC32900), the Tianjin Education Commission Research Project (grant no. 2018KJ043,20140118), the Tianjin Education Commission Research Project (grant no. 2018KJ045), and the Tianjin Science and Technology Planning Project (no. 20YFZCSY00060).Tianjin Municipal Health Commission Youth Project (grant no. TJWJ2021QN001);Medjaden Academy & Research Foundation for Young Scientists (Grant No. MJR20221011);Tianjin Key Medical Discipline(Specialty) Construction project(Grant TJYXZDXK-028A).

## Conflict of interest

The authors declare that the research was conducted in the absence of any commercial or financial relationships that could be construed as a potential conflict of interest.

## Publisher’s note

All claims expressed in this article are solely those of the authors and do not necessarily represent those of their affiliated organizations, or those of the publisher, the editors and the reviewers. Any product that may be evaluated in this article, or claim that may be made by its manufacturer, is not guaranteed or endorsed by the publisher.
